# Nullifying phosphatidic acid effect and controlling phospholipase D associated browning in litchi pericarp through combinatorial application of hexanal and inositol

**DOI:** 10.1038/s41598-019-38694-5

**Published:** 2019-02-20

**Authors:** Bharat Bhushan, Satish Kumar, Manoj Kumar Mahawar, Kirti Jalgaonkar, Ajinath Shridhar Dukare, Bhushan Bibwe, Vijay Singh Meena, Narender Negi, Rajesh Kumari Narwal, Ajay Pal

**Affiliations:** 1 Horticultural Crop Processing Division, ICAR-Central Institute of Post-Harvest Engineering and Technology, Abohar, 152 116 India; 2 School of Drought Stress Management, ICAR- National Institute of Abiotic Stress Management, Malegaon, Baramati, 413115 India; 3 Department of Biochemistry, College of Basic Sciences and Humanities, CCS Haryana Agricultural University, Hisar, 125 004 India

## Abstract

The role of phospholipid modification initiated by phospholipase D (PLD) in enzymatic browning has been revoked through this study. Various alcohols and aldehydes were tried to read out their PLD controlling behaviour. Based on *in-vitro* results, reagents like hexanal and inositol were used to regulate PLD activity of litchi fruit stored at ambient temperature and their effects on fruit quality and physiological characteristics were also investigated. The results showed that combinatorial chemical treatment was successful in maintaining freshness of fruit through delayed physiological loss in weight and hence maintaining firmness. Combinatorial treated fruit had lower browning index than control by day 7. This novel treatment also maintained comparable levels of total phenolics and lowered the level of malondialdehyde. Evaluation of antioxidative enzymatic profile also confirmed the alleviation of oxidative stress of litchi fruit at ambient temperature. Thus, this strategy of enzyme regulation could play a vital role in overall quality maintenance of litchi fruit.

## Introduction

Litchi fruit is known for its color, flavour and texture which make its crop of high commercial value. The respiratory physiology of this fruit is of non-climacteric type which doesn’t show any spurt in response to exogenous ethylene. Apart from this, it does not attain its quality attributes if it is harvested before physiological maturity. Thus, commercial maturity of this fruit must be taken into consideration for harvesting. The morphological parameters like color and tubercles on fruit surface may be used to decide the harvesting stage. For distant market biochemical parameters like 16–18 °Bx TSS (total soluble solids) and 0.3 to 0.4 per cent acidity has been proved to be significant in deciding the harvesting stage^1^.

The commercial success of litchi fruit in global market is limited by few factors. First, it is available only for a very short period of time due to its narrow genetic base and restricted climactic conditions. Second is the post-harvest problem of this fruit which is the most pressing problem in the tropical countries like India. The storage behaviour of this fruit also depends upon local environment and cultivar especially fruit axial dimensions, peel diameter or stone size^2^. At ambient temperature, post-harvest management personnel allocate this fruit to a perishable category of fruit with a shelf life of 2–3 days. The predicted post-harvest loss of this fruit may rise up to 50 per cent prior to its consumption^3^.

Since appearance of fruit is an appealing factor in purchase and consumption by consumer, color or its related quality attributes are of major concern for the stakeholders in this crop. Fruit browning is commonly observed after 1 day of storage at >30 °C. The pericarp browning initially starts from the protuberances and then slowly spreads over the entire surface of pericarp, if not controlled. Loss of water from fruit surface, oxidation of biomolecules, electrolyte leakage and anthocyanin degradation are the major known factors for fruit browning.

It is presumed that all surface based storage disorders in color and texture are membrane originated but the sequence starting from origin to terminal site of membrane injury and the underlying mechanism has not yet been dissected. Preservation of membrane structure is helpful in maintaining fruit quality and lengthening shelf-life^4^. In the existing literature on post-harvest biochemistry of fruit pericarp, role of key enzymes like phospholipase D (PLD) in addition to phenylalanine ammonia lyase (PAL), polyphenol oxidase (PPO) and peroxidase (POD) has always been suspected. Molecular studies and metabolomics have revealed the versatile role of PLD in fruit development, ripening and desiccation-browning where enzyme is directly involved through regulatory network of enzymes or indirectly through signalling mechanism of phospholipid metabolism^5^. In response to water deficit, induction of PLD is now a well-known response^6^.

Sulphur fumigation is most commonly used for color preservation of litchi fruit but benefits of color preservation through sulphite gas may be outweighed by disadvantages such as reduced fruit quality, health issues and environmental concerns. Cold storage of litchi fruit increases its shelf-life up to 30 days for extended availability beyond its season but once the fruit comes out to ambient conditions, it gets deteriorated faster than the non-cold stored^7^. Numerous tactics have been devised and applied to this fruit to prevent browning. Out of those, use of enzyme inhibitors looks very promising due to its specificity and efficacy. Apart from this, controlling biochemical reactions by biochemical means seems to be more appropriate than by any other means. Efficacy of various specific and non-specific inhibitors have been investigated in various fruits^4,8,9^.

Lipid peroxidation of pericarp is one of the major causes of browning which not only changes the permeability of pericarp for water loss but also leads to loss of fluidity and overall functionality. Both the losses may lead to brown spots on the fruits and cracks spread all over the surface. Since PLD comes first in sequence or cascade of events associated with browning or stress attack and it is the rate limiting step of the pathway, it appears to be the regulator in the flux of metabolites through membrane bottleneck. These facts indirectly denote that controlling the PLD activity can be critical for enhancing the shelf-life and quality preservation of fruits and vegetables. The enzymatic action of PLD on membrane phospholipids removes a head group present at the third carbon in ester position and forms an intermediate transition complex of enzyme and the modified substrate. Finally, completion of the enzymatic reaction generates phosphatidic acid which acts as second messenger and carries the signal of membrane alteration and destruction downstream of the protein cascade. The expression level of PLD differs in different cultivars and at different developmental stages of fruit. Sub-cellular fractionation of pericarp may also give differential level of PLD activity^10^. Inhibition of its activity by various treatments can possibly check the degradation of phospholipids in membranes and may result in inhibition of browning reaction and improvement of fruit quality.

Hexanal and inositol are aldehyde and alcohol based organic compounds which are synthesized by the plants for aroma and sequestration of minerals. Hexanal is off-shoot of lipid metabolism whereas inositol is a metabolite of carbohydrate metabolism. Both are considered under GRAS category of compounds for food industry. In the previous studies, a number of attempts have been made to alleviate browning problem on the basis of antioxidants or maintenance of moisture. One recent example is the usage of benzyl-amino-purine where level of lipid peroxidation has been minimized without stating the molecular mechanism, although control of browning has been liked to reduced ROS production^11^. A meagre attention has been paid to understand the integrated mechanism of browning and its control by various means. The present combinatorial approach of PLD inhibition by hexanal and enhancement of reductive state using antioxidants like inositol would not only reduce the phospholipid degradation but also the downstream oxidative processes^5^. Inositol serves dual purposes of antioxidant augmentation and metal sequestration for controlling miscellaneous browning enzymes. However, the stage of application of enzyme inhibitors is critical since PLD inhibition has very little effect once the membrane deterioration is accelerated.

## Materials and Methods

### Chemicals

Phosphatidyl choline, PLD, choline chloride and other phospholipids were procured from MP Biomedicals, India. All other chemicals and reagents were purchased from Sisco Research Laboratories Pvt. Ltd. India.

### Sampling procedure

Litchi c.v. *culcuttia* fruits were harvested at optimum physiological maturity from the orchard of Regional Research Station, Gurdaspur, Punjab Agricultural University campus during June 2014. The fruits of homogeneous color and size free from any visual injuries were selected for experimental trials. A large batch of fruits was used to analyze properties at harvesting time and remaining fruits were split into batches for treatment and sampling. Fruits dipped in water at 25 °C were treated as control. Reagents like inositol/glycerol with hexanal (0.01%) were used at 1% concentration of for 15 minutes at 25 °C. After combinatorial treatment, litchi fruits from each combination were air-dried, randomly divided into different lots and stored in a polyethylene bag with optimized ventilation or LDPE at 25 °C. Both treated and control fruits were taken out at regular intervals for analysis up to one week of storage.

### Physico-chemical parameters

A total of 20 fruits were selected in replication from each treatment and evaluated for physiological loss in weight, titrable acidity and TSS content. Pericarp browning was assessed by measuring the extent of total browned area on fruit pericarp. Browning index grading was prepared according to the method of Jiang (2015) and firmness was checked using texture analyzer^12^. Browning index of 3.0 was considered as unsalable fruit. For firmness analysis, the probe head used was cylindrical with 5 mm penetration and the applied force was 50 N. The crosshead speed was 50 mm min^−1^. Data acquisition was 100 points per sec. Probe initial height was adjusted to 50 mm from the equatorial positions of the fruit.

### Membrane leakage

Litchi fruits were peeled and their pericarp discs (including endocarp) of 1.2 cm diameter were made. These discs from each fruit were weighed and placed in a beaker containing 25 mL distilled water. The discs were then transferred to a beaker containing 30 mL of 0.3 M mannitol solution at 25 °C with shaking for 30 min. Electrical conductivity (EC) of the sample with and without incubation were measured. Membrane leakage was calculated as EC_s_/EC_c_ × 100.

### Determination of antioxidative potential

Litchi pericarp tissues (2 g) were minsed in 10 mL of 0.1 M potassium phosphate buffer (pH 8.0) for the extraction of soluble antioxidant molecules. The antioxidant activity was expressed using DPPH radical scavenging activity and measured according to Shimada *et al*. (1992) with slight modifications^13^.

### Browning substrates and enzymes associated with membrane system

Fruit pericarp (2 g) was crushed in extraction buffer as stated above and homogenate was centrifuged at 19,000 × g for 20 min. The activities of lipid-associated enzymes such as PLD^14^, PAL^15^ and PPO^16^ was determined in supernatant. All the enzyme assays were carried out at refrigerated temperature. Total phenolic compounds were measured by Prussian blue assay.

### Statistical analysis

Selected data are presented as means of at least three independent experiments (n = 3), each selected experiment had a minimum of three replicates of each sample. Standard error of mean was taken into consideration while depicting the error bars in each figure. Significant and insignificant differences were noted according to the independent sample t-test for each storage period.

## Result and Discussion

Pericarp browning of litchi fruit depends upon storage temperature. At ambient temperature, pericarp started to turn brown after one day whereas it took three weeks to show symptoms of browning at cold storage (data not shown). The comparative color retention was quite evident from the more positive ‘a’ values in the Hunter color lab system and was also better in litchi fruits stored at cold storage (data not shown; Supplementary Info 1 shown).

Anthocyanin pigments, which impart red color to litchi fruit, are highly sensitive to temperature. Zheng and Tian (2006) found that oxalic acid delayed browning and suggested its use after harvest^17^. Use of butanol and hexanal has been made on litchi fruits after its harvest to control oxidative stress^4,18^.

**Figure 1:**
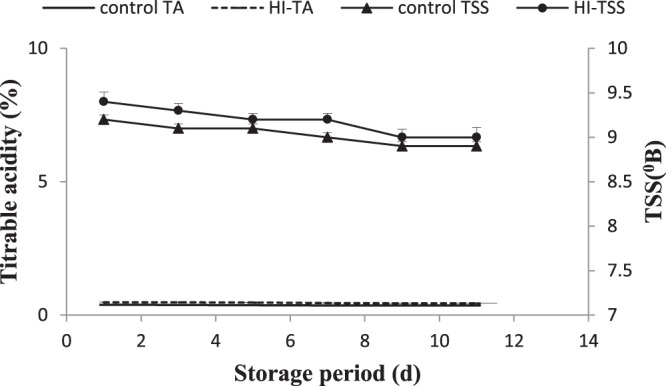
Changes in titrable acidity and TSS of litchi pulp under formulation treatment. The significant and insignificant differences according to the independent sample t-test (P < 0.05 and P < 0.01, respectively) for each storage period was noted. TA: titrable acidity; TSS: Total soluble solids; HI-hexanal-inositol.

A slight change in total soluble solids (TSS) and titrable acidity (TA) in pulp of treated whole fruit sample was observed during storage at 25 °C (Fig. 1). In contrast, average values of both parameters in control samples tended to be lower than treated samples at the same temperature. TSS and TA often reflect the eating quality. Jiang and co-workers (2004) reported that TSS and TA contents decreased during storage at low temperature^19^.

**Figure 2:**
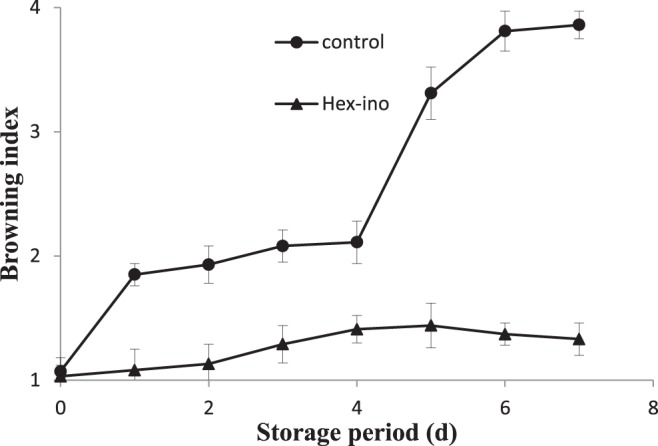
Assessment of magnitude of pericarp browning in control and treated samples. The significant (5, 6, 7 DAS) and insignificant differences (1, 2, 3, 4 DAS) according to the independent sample t-test (P < 0.05 and P < 0.01, respectively) for each storage period was noted.

Combinatorial application of inositol and hexanal was found effective in minimizing 3% decrease in weight loss. This moisture loss also revealed that control fruit has a much faster rate of browning rendering the fruit unmarketable. Control fruit has a browning index of 4.0 after 5 days of storage (Fig. 2) while application of inositol and hexanal led to fruit with least browning index which remained nearly one in the first six days of treatment.

**Figure 3:**
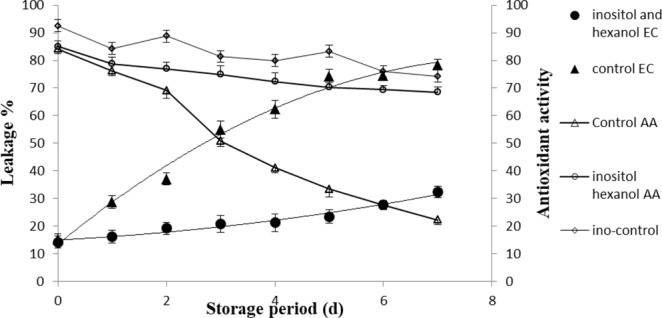
Electrolyte leakage from the litchi pericarp and its antioxidant activity. The significant and insignificant differences according to the independent sample t-test (P < 0.05 and P < 0.01, respectively) for each storage period was noted. AA-antioxidant activity; EC-Electrolyte conductance.

Water loss is an important attribute that contributes to the qualitative and quantitative status of the produce. In fruit, moisture loss takes place from the peel of fruit which leads to the development of micro-cracks in the pericarp. These cracks in conjunction lead to a continuum for more intense release of moisture from the mesocarp. The electrolyte leakage from the pericarp also appears to be higher in cracked skin or pericarp. Exactly, the same happened in our experiment where the pericarp leakage in control fruits tended to remain higher as compared to treated samples at the initial storage days and increased exponentially at later stages (Fig. 3). Membrane permeability of control pericarp also increased increased with storage time but decreased in treated samples after two days of treatment. The results suggest that membrane integrity is severely compromised in control samples whereas the treated samples have substantially prevented themselves probably by transphosphatidylation.

Loss of water is the hallmark of deteriorating quality of litchi fruit. Jiang and Fu (1999) found that loss of weight of fruit packed in polyethylene bags was mainly due to respiration and storage at low temperature slowed this respiratory loss^20^. The action mechanism of catechin like pyrogallol in preventing the intense water loss appears to be through lowering respiration rate and membrane permeability^21^.

**Figure 4:**
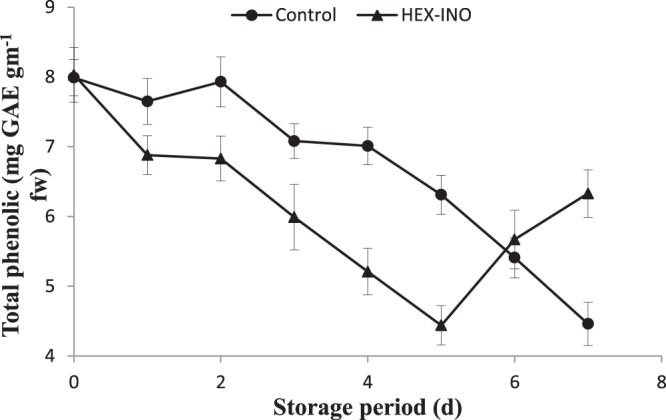
Effect of treatment on the total phenolic content. The significant and insignificant differences according to the independent sample t-test (P < 0.05 and P < 0.01, respectively) for each storage period was noted.

Polyphenolic contents initially increased slightly and then declined during storage at ambient temperature (Fig. 4). In contrast, the polyphenolic contents tended to decrease in treated samples and increased after 6 days of treatment. On other hand, DPPH activity in treated samples remained constant as compared to control fruit on all the storage days at 25 °C (Fig. 3). Results suggest that membrane integrity has been preserved in the presence of inositol and hexanal. The content of polyphenolics in the pericarp varies with the development stages. However, the PPO/POD enzyme based degradation (specifically oxidation reactions) increases after attainment of maturity resulting in pericarp browning. The hydrolytic products of phenolic compounds act as possible substrates for POD and PPO^22^. Therefore, inhibiting the degradation of phenolic compounds or stimulating the synthesis of anthocyanins through PAL may be a possible strategy to prevent browning or establishing the red color of the litchi^21,23,24^.

It is always presumed that antioxidant capacity of bright colored fruits and vegetables is more than their lesser colourful counterparts. This antioxidant capacity could be enzymatic or non-enzymatic^24,25^. In this study, a significant (p < 0.05) correlation was observed between concentration of phenolic compounds and antioxidant activity of litchi fruit during storage. The internal antioxidant competence was enhanced by addition of inositol and phenolic content also drastically increased from 5^th^ to 7^th^ day of treatment (Figs 3 and 4). The inositol with specified hydroxylation prevents the iron mediated enzyme catalysed lipid or ascorbic acid oxidation and fortifies the endogenous competence^26^.

**Figure 5:**
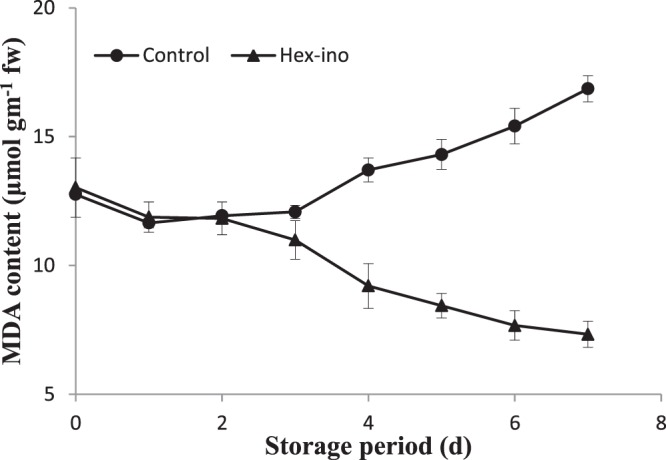
Assessment of membrane damage through MDA estimation. The significant and insignificant differences according to the independent sample t-test (P < 0.05 and P < 0.01, respectively) for each storage period was noted.

MDA content in pericarp increased in both the samples with extended storage time (Fig. 5). However, a lesser increase in MDA content in treated pericarp samples was observed as compared to control fruit pericarp. The results indicate that inositol in conjunction with hexanal could dramatically help in preventing the membrane lipid peroxidation in the pericarp of harvested litchi fruit. MDA, as a marker of membrane lipid peroxidation, could be used to evaluate the degree of membrane lipid peroxidation^27^. Its concentration reflects the extent of membrane lipid peroxidation caused by oxidation^28^. Loss of membrane integrity leads to de-compartmentation of enzymes and substrates which are involved in pericarp browning. The relative leakage rate is an important index of membrane integrity. Shelf life property has been explored in the form of textural properties such as bioyield point and pulp firmness which dictate the freshness and saleability of fruit. As compared to control, the bioyield point and firmness were significantly (p < 0.01) higher in combined hexanal and inositol treatment (Table 1). Similar results with hexanal has earlier been reported^4^. The mechanism of action of a reported head group like cyclic C6-inositol based PLD inhibitor is the same.

**Table 1:** Effect of various treatments on bioyield point and pulp firmness of litchi at different storage period.

Days after storage	Bioyield Point (g)					Flesh or pulp Firmness (g)				
	1	2	3	4	5	1	2	3	4	5
Control	1152 ± 25	951 ± 14	691 ± 14	272 ± 21	44 ± 5	411 ± 9	383 ± 8	323 ± 5	222 ± 4	107 ± 2
Hexanal	1158 ± 28	1008 ± 17	797 ± 7	273 ± 9	46 ± 4	434 ± 9	387 ± 7	358 ± 5	225 ± 4	147 ± 3
Inositol	1244 ± 10	1097 ± 20	799 ± 20	284 ± 26	233 ± 7	477 ± 15	388 ± 7	359 ± 4	299 ± 4	154 ± 3
Hex-inositol	1320 ± 30	1115 ± 10	882 ± 10	436 ± 13	265 ± 3	582 ± 19	396 ± 6	367 ± 4	311 ± 3	215 ± 3

Selected data are presented as means of at least three independent experiments (n = 3), each selected experiment had a minimum of three replicates of each sample.

**Figure 6:**
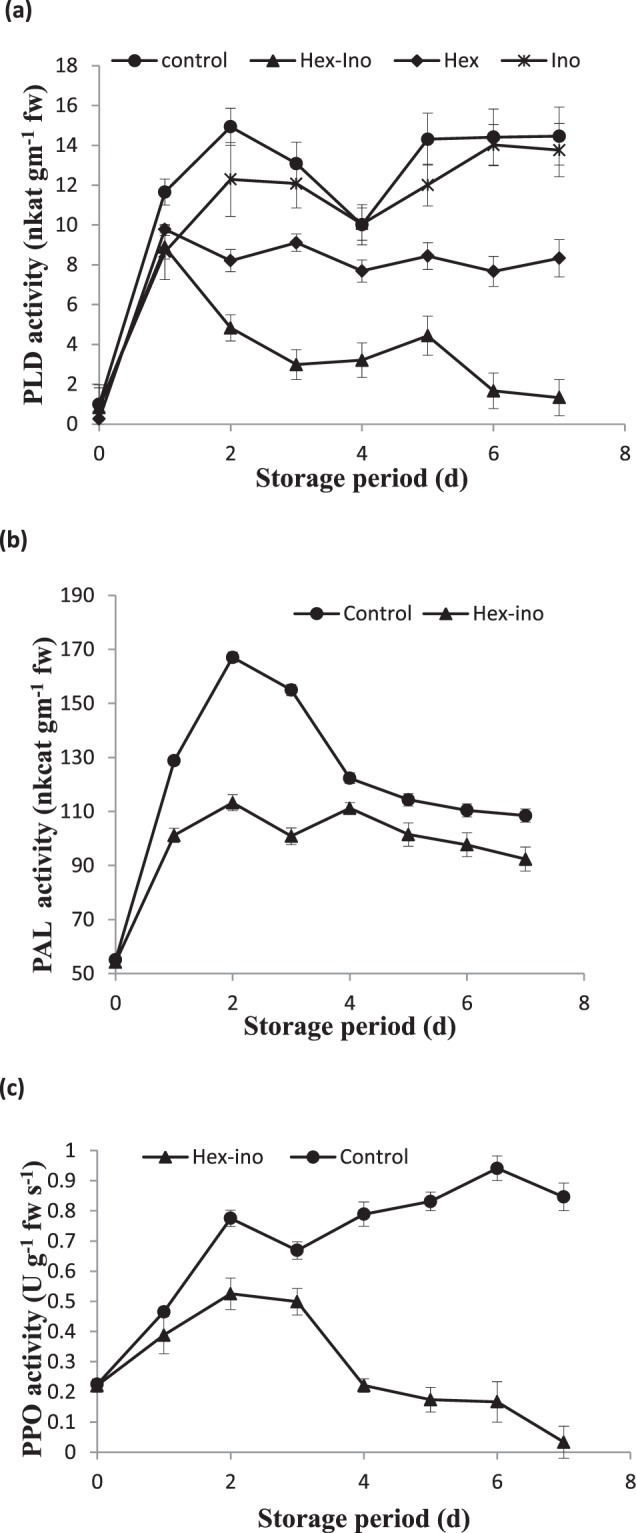
Profiling of browning causing or browning associated enzymes. The significant and insignificant differences according to the independent sample t-test (P < 0.05 and P < 0.01, respectively) for each storage period was noted.

Browning associated three key enzymes namely PLD, PAL and PPO were studied. As shown in Fig. 6a, the PLD activity of control fruits started to increase from the 1^st^ day of storage and reached its peak on 2^nd^ day. On 3^rd^ day, it started to decrease till the 4^th^ day and afterward again started to rise. The treated pericarp, however, has lower PLD activity throughout the storage period. The extent to which different treatments are effective could be visualized through lower PLD activity from the 2^nd^ day. Although inositol was as good as hexanal yet in combination it controlled the enzyme activity to a better extent. Combination treatment of chitosan and salicylic acid and its synergistic effect has earlier been reported^29^.

The activity of wounding enzyme phenylalanine ammonia lyase (PAL) started to rise from 1^st^ day of experiment till 3^rd^ day and thereafter it declined in control and treated pericarp (Fig. 6b). The activity level of PAL was lower in treated samples. PPO activity displayed unusual pattern from 3^rd^ day to 6^th^ day of storage in control whereas showed lower activity from 3^rd^ day of storage to 7^th^ day of storage (Fig. 6c).

The antioxidant profile of any food commodity is generally dictated by vitamins A, C and E. Apart from these, enzymes like SOD, glutathione peroxidase, ascorbate peroxidase etc. constitute the enzymatic antioxidant system. Moreover, anthocyanin pigment is another antioxidant molecule in litchi and PAL is the key enzyme involved in its biosynthesis. Zhang *et al*. (2005) reported that degradation of anthocyanin led to formation of anthocyanidin which is further degraded by POD and PPO and resulting in fruit browning^30^. Activation of PAL and inhibition of PPO/POD through the application of pyrogallol on litchi fruit led to higher average concentrations of anthocyanins and phenolics. The effect of alcohols on wounding enzyme like PAL is not at post-translational level but either at transcriptional or post-transcriptional level^7^.

PLD belongs to the class of phosphohydrolases and generally found in mitochondrial membranes, microsomal membranes and cytosol. It can also hydrolyse a variety of head groups like inositol, glycerol, choline etc. present in the phospholipids. Depending upon the stage of tissue or storage conditions, PLD migrates from membrane to cytosol or cell wall space (at ripening stage). This enzyme is found, to a considerable level, in the cytosol of unstressed cells. Phosphatidic acid, diacylglycerol and free fatty acids released through the sequential enzymatic actions of PLD, PAP*ase* and non-specific acyl-hydrolase are known markers for membrane damage. The phosphatidic acid produced under stress condition acts as second messenger to stimulate the chain of free radical production^31^.

**Figure 7:**
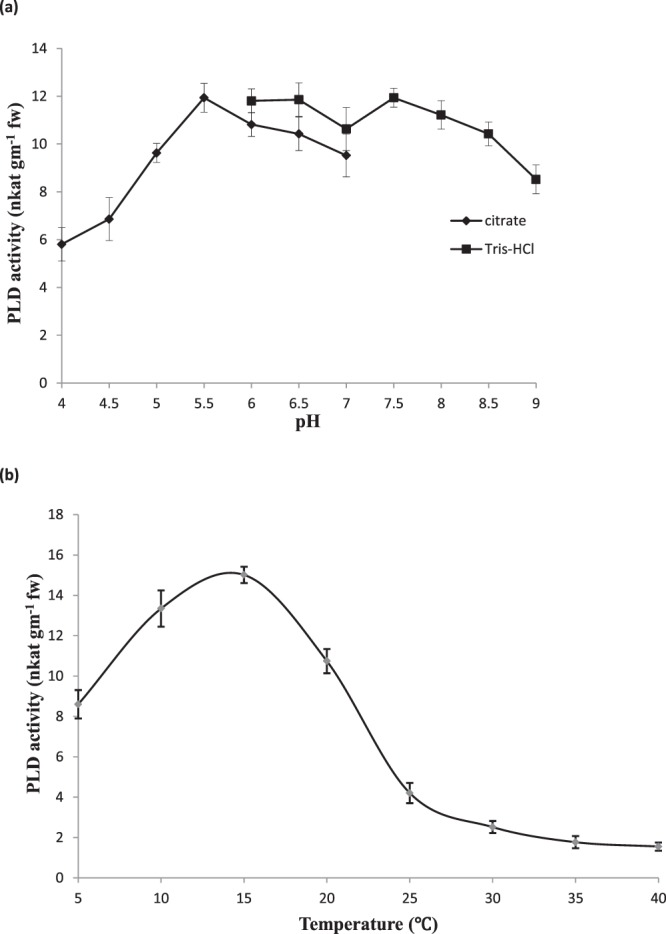
Effect of pH (**a**) and temperature (**b**) on PLD activity of commercial enzyme. PLD activity was assayed at various pH values using Tris-HCl and citrate-phosphate buffer. PLD activity was assayed after equilibration of assay mixture at a given temperature for 10 minutes. Reaction was started then and allowed for 10 minutes for release of choline.

Simulation studies of enzyme inhibition technology were also executed using enzyme and various chemicals for different reaction parameters. Under fruit processing conditions or uncongenial storage atmosphere, loss of moisture associated cracking/abrasion causes wounding of the tissue which results in changes in *p*H by mixing of intracellular contents. Therefore, the potential effect of change in *p*H on cytosolic PLD was investigated. Citrate buffer in the *p*H range 4.0–7.0 and Tris-HCl in the *p*H range 6.0–9.0 were used in these estimations (Fig. 7). Cytosolic preparation have showed enhanced activity at the *p*Hs 5.5 and 7.5 indicating a broad *p*H optima of PLD. Observation indicates that maintaining an acidic *p*H will not only help in red color restoration but also control the enzyme activity to some extent.

Shelf-life of perishables depends on the deficit between fruit temperature and local storage temperature. So the effect of temperature on PLD activity was studied, *in vitro*, to understand the role of PLD in membrane dynamics at high temperature. It was found that PLD remains active at low temperature and its activity starts to decline at a temperature above 20 °C (Fig. 7). Higher temperature can inactivate the enzyme but with increased temperature, peel or pericarp starts losing moisture and gets brittle. Hence, a delicate balance of temperature and moisture content is necessary to control the activity of PLD.

**Figure 8:**
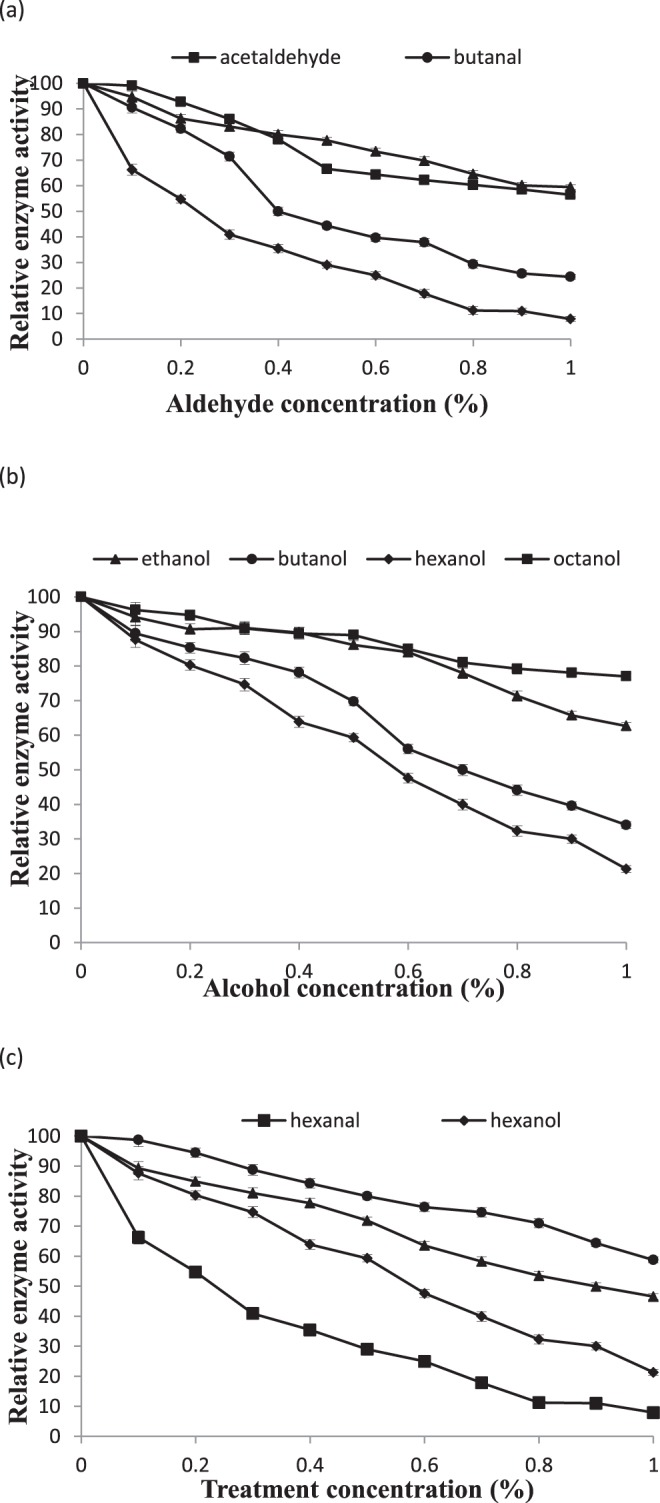
Effect of C_2_ - C_6_ aldehyde (**a**), C_2_ - C_6_ alcohol (**b**) and concentration of selected C_6_- aldehyde/alcohols (**c**) on PLD activity of commercial enzyme. PLD activity was assayed after adding the appropriate amount of reagents in basic assay mixture and equilibration of assay mixture at optimal temperature and pH for 10 minutes. Reaction was started then and allowed for 10 minutes for release of choline.

The efficacy of alcohols and aldehydes of different chain lengths was assessed for PLD inhibition. Methanal (small chain aldehyde) and methanol (small chain alcohol) effectively inhibited PLD enzyme but were not further tried because of their toxicity. It was also found that with increase in carbon length there was a gradual increase in PLD inhibition (Fig. 8a,b). Acetaldehyde and ethanol are released during over-maturation of fruit during storage at low temperature and low oxygen environment. Therefore, their effect was explored on PLD activity. These reagents marginally stimulated the enzyme activity at lower concentrations (<0.2%) but inhibited at higher concentrations (>0.2%). So, the endogenous production of acetaldehyde may supplement the inhibition process initiated by exogenous alcohol or aldehyde. Previously reported chemicals like butanol and butanal were also used and found inhibitory. Overall, small-chain alcohols and aldehydes which are the normal metabolites in perishables could affect the membrane deteriorative processes.

Production of long-chain aldehydes such as hexanal and hexenal, and alcohols such as hexanol and hexenol is a normal event during senescence or wounding where LOX enzyme follows the PLD initiated signal cascade. Hexanal and hexanoic acid are common components of flavor volatiles in ripening fruits. Because of their natural occurrence and abundance in fruits and vegetables, the effect of hexanal and hexanol on PLD activity was investigated (Fig. 8c). It was observed that aldehydes are more effective than alcohols. The efficacy of –ane or –ene moieties was assessed by comparative evaluation of the effects of hexenal and hexanol on PLD enzyme (Fig. 8c) where alkanes were found better inhibitors than alkenes. These results point out the natural regulation of PLD activity in healthy tissues by endogenous volatiles and similar mechanisms. Thus, inhibition of PLD by hexanal and hexanol could serve as a checkpoint for arresting membrane lipid degradation. There are natural by-products like peel which produces these metabolites and/or have diverse array of polyphenolics or isoprenoids which could be of potential use in preventing excessive membrane lipid degradation during storage or under processing conditions.

**Figure 9:**
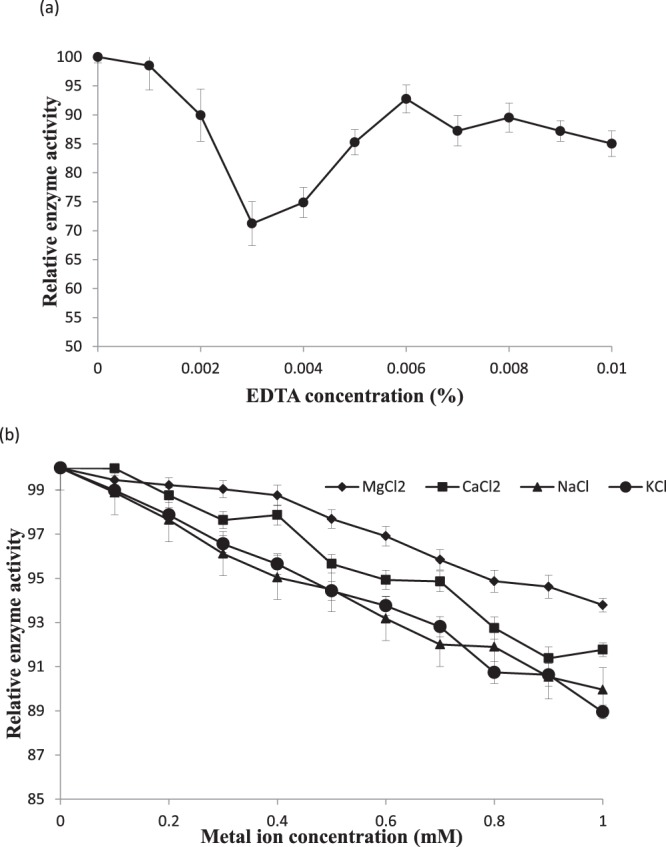
Effect of EDTA (**a**) and metal ions (**b**) on PLD activity of commercial enzyme. PLD activity was assayed after equilibration of assay mixture at optimal temperature and pH for 10 minutes. Reaction was started then and allowed for 10 minutes for release of choline.

Fruits and vegetables are known for their mineral strength which comprises potassium, magnesium, calcium, phosphorus etc. Upon decompartmentalization, these minerals may have strong impact on various cellular activities catalysed by enzymes. Specific assays were conducted to investigate the potential effect of various mineral/metal ions on PLD activity. Monovalent or divalent ions did not have significant effect at concentrations lower than 0.1 mM (p < 0.01; not shown). In fact, PLD has a requirement for metal ions (monovalent or divalent) so chelation with EDTA (0.01%) led to control of activity but concentrations above 0.03% had no significant (p < 0.01) effect in inhibition process (Fig. 9).

Previous reports have stated that an increase in sub-micromolar level promotes the enzyme targeting to membrane for substrate utilization which are amply available in the membrane. An accumulated calcium ions in cytosol and induced PLD activity with simultaneous increase in ATP*ase* activities of vacuolar and plasma membrane sites (H^+^-ATP*ase*) have been observed during stress or senescence^32^. When the stimulation/inhibition by magnesium and calcium ions was compared, the effect of magnesium ion was slightly higher than calcium ions. There was no substantial effect of monovalent ions on the PLD preparation.

**Figure 10:**
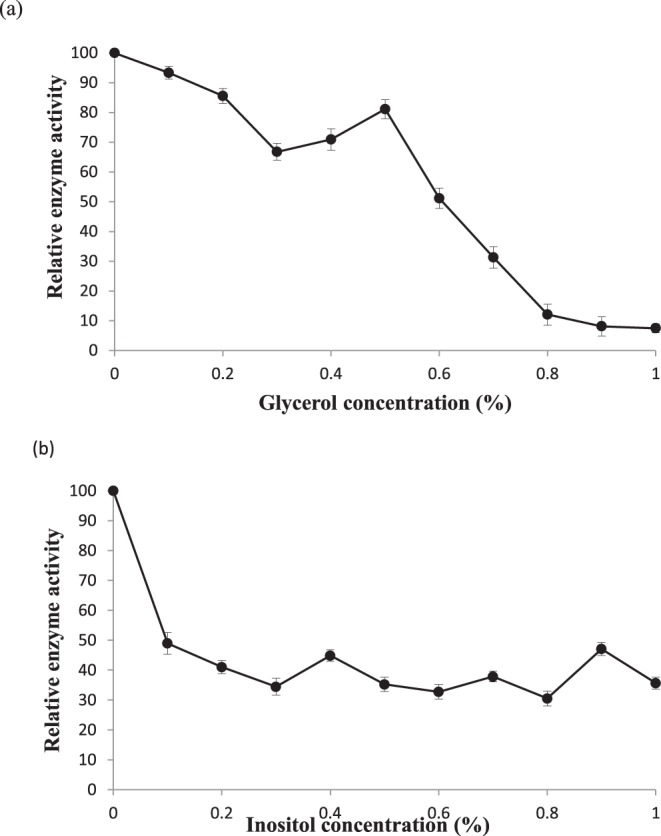
Effect of glycerol (**a**) and inositol (**b**) on PLD activity of commercial enzyme. PLD activity was assayed after adding the appropriate amount of reagents in basic assay mixture and equilibration of assay mixture at an optimal temperature and pH for 10 minutes. Reaction was started then and allowed for 10 minutes for release of choline.

Finally, two reagents which were used in *in-vivo* experimental trials were used for *in-vitro* trials. Selection of the head group reagents like glycerol and inositol was based upon the transphosphatidylation reaction which is an integral part of PLD reaction mechanism. Glycerol was found more effective than inositol in controlling PLD activity. At 1%concentration, glycerol controlled up to 90% activity whereas inositol could control by 60% (Fig. 10). Transphosphatidylation with these reagents may lead to lesser production of phosphatidic acid which is known for its ill-effects like membrane deterioration (Supplementary Info. 2).

## Conclusion

PLD action has a profound effect on pericarp and makes visible the membrane deterioration. Its activity can be controlled through its transphosphatidylation reaction through various phospholipid head groups. If pericarp is dipped in alcohol or aldehyde based solvent, trans-phosphatidylation reaction of PLD may transfer alcohol to phosphatidic acid and limit the production of phosphatidic acids. Trans-phosphatidylation using inositol is the siphoning-off reaction for phosphatidic acid and use of hexanal is the inactivating approach for PLD. Both, in combination, could be used as a tool to control pericarp browning and membrane degradation. Apart from it, slowing down the free radical production and lesser lipid peroxidation as depicted by antioxidant capacity and MDA values, respectively has helped in the maintenance of membrane system. The strategyworks against the ill effects of membrane decompartmentalization.

## Supplementary information

Supplementary Information 1,2
