# Supplementary material for: Nullifying phosphatidic acid effect and controlling phospholipase D associated browning in litchi pericarp through combinatorial application of hexanal and inositol
Source: Sci Rep. 2019 Feb 20;9:2402. doi: 10.1038/s41598-019-38694-5 (PMC6382881; doi:10.1038/s41598-019-38694-5)
Supplement: Supplementary file 1 — Supplementary Information 1,2 [file 41598_2019_38694_MOESM1_ESM.pdf]

Nullifying phosphatidic acid effect and controlling phospholipase D associated browning in litchi pericarp through combinatorial application of hexanal and inositol

Bharat Bhushan, Satish Kumar, Manoj Kumar Mahawar, Kirti Jalgaonkar, Ajinath Dukare, Bibwe Bhushan, Vijay Singh Meena, Narender Negi, Rajesh Kumari Narwal and Ajay Pal

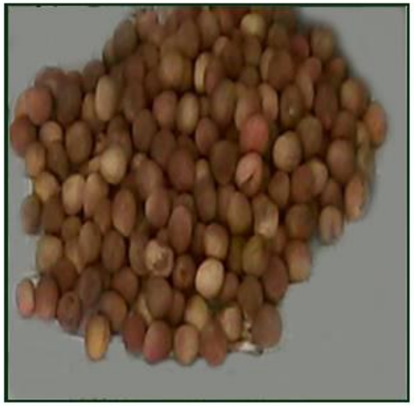

INOSITOL

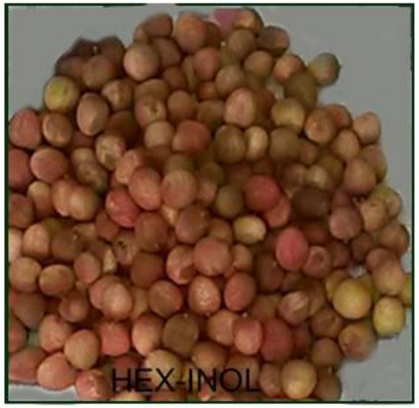

HEX-INOL

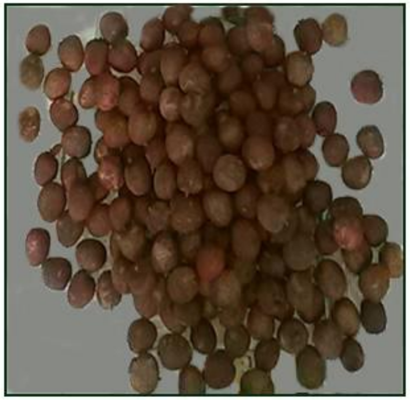

CONTROL

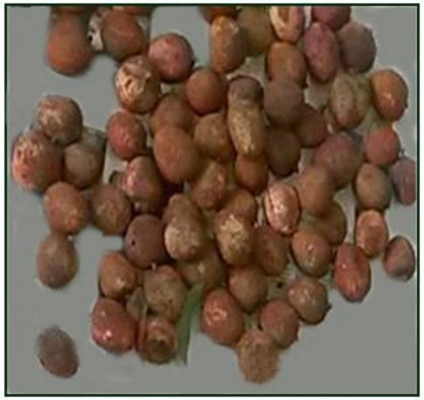

HEXANOL

Supp. Fig 1: Control of browning in litchi using enzyme inhibitors. The combinatorial treatment of hexanal (0.01%) and inositol (1%) in litchi (c.v. culcuttia) and thereafter storage at 25°C has helped in preservation of the pericarp color and shelf-life till 7th day of storage.

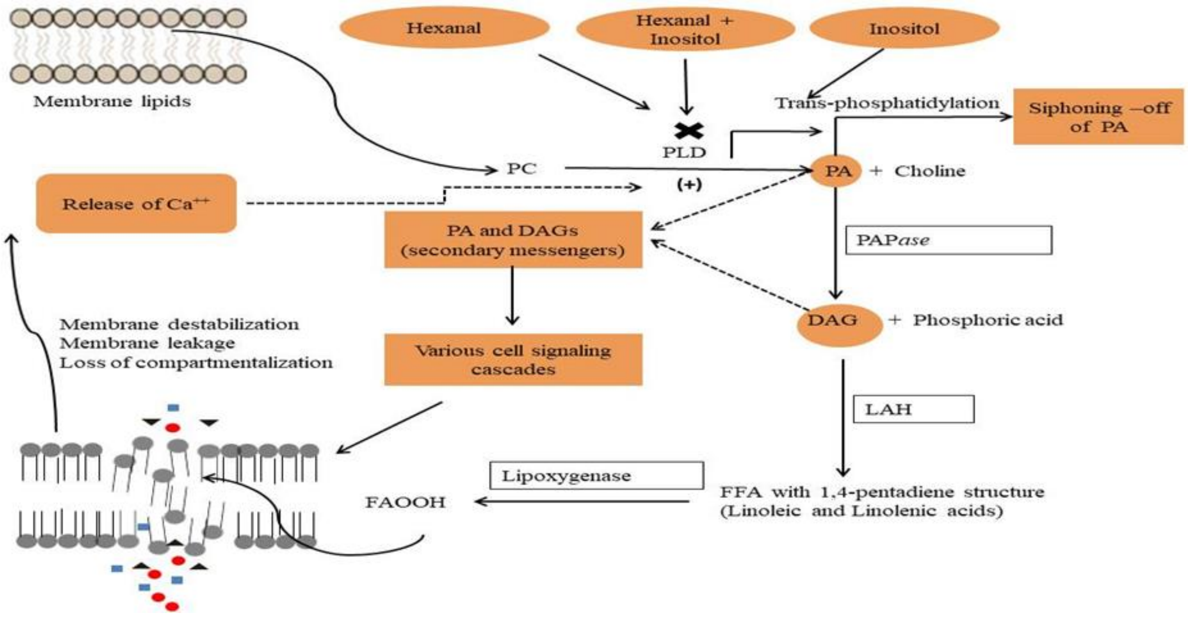

Supplementary Fig 2 - The schematic representation of different enzymatic reactions involved in membrane deterioration resulting in physicochemical changes in the pericarp of the Litchi fruit. The various abbreviations used represent FFA (Free fatty acids), FAOOH (hydroperoxide products of free fatty acids), PLD (Phospholipase D), PAPase (Phosphatidate phosphatase), LAH (Lipolytic Acyl Hydrolase), PA (Phosphatidic acid), DAG (Diacylglycerol), Phosphatidyl Choline. The bold line arrow depicts the crucial enzymatic reactions and their direct control whereas dotted line arrow depict the cascade of events involved in signal transduction.
